# mHealth Applications in the Assessment of Musculoskeletal Pain in Menopausal Women: Protocol for a Scoping Review

**DOI:** 10.12688/hrbopenres.14515.2

**Published:** 2026-09-22

**Authors:** Olivia Buttenham, Karen McCreesh, Katie Crowley

**Affiliations:** 1 Faculty of Education and Health Sciences, University of Limerick School of Medicine, Limerick, County Limerick, Ireland; 2 Faculty of Education and Health Sciences, University of Limerick School of Allied Health, Limerick, Limerick County, Ireland; 3 University of Limerick Health Research Institute, Limerick, County Limerick, Ireland; 4 Lero, the Research Ireland Centre for Software, University of Limerick Department of Computer Science and Information Systems, Limerick, County Limerick, Ireland; 5 University of Limerick Department of Computer Science and Information Systems, Limerick, County Limerick, Ireland

**Keywords:** Menopause; Musculoskeletal symptoms; mHealth; Symptom tracking; Self-management; Scoping review; Study protocol.

## Abstract

**Background:**

Menopause is a major life transition with diverse physical, psychological, and social symptoms. More than 70% of women experience musculoskeletal (MSK) pain during the menopausal transition, and around 25% are disabled by it; this symptom cluster was recently characterised as the musculoskeletal syndrome of menopause. These symptoms are frequently normalised as an inevitable consequence of ageing rather than recognised as treatable, contributing to underdiagnosis and limited management. Mobile health (mHealth) applications increasingly deliver education, symptom tracking, and self-management support to menopausal women, yet the evidence base is fragmented, and how well this research captures MSK symptoms, rather than leaving them embedded within broader symptom measures, remains unclear.

**Methods:**

This scoping review will follow JBI methodology and be reported using the PRISMA Extension for Scoping Reviews (PRISMA-ScR). Six bibliographic databases (Embase, Web of Science, Scopus, PubMed/MEDLINE, CINAHL, and IEEE Xplore) will be searched, with reference-list hand-searching of included studies. Records will be screened in duplicate in Covidence; title/abstract screening will retain records indicating symptom monitoring or tracking with a potential MSK link (including composite menopause symptom measures) or strength-oriented exercise, with MSK relevance confirmed at full text. A structured charting form will capture intervention characteristics, symptom-tracking functionality, study design, and outcomes, alongside an MSK-directness classification distinguishing direct measurement from indirect coverage (within composite measures, or exercise/physical-activity components). Reported acceptability, usability, and engagement will be organised through the Technology Acceptance Model. Findings will be synthesised narratively using tables and thematic summaries.

**Conclusions:**

This review will map how published, menopause-related mHealth research represents MSK symptoms, and whether they are assessed directly or only within broader symptom measures, rather than evaluating the applications themselves, which are the focus of a separate, complementary review. Results will be submitted to a peer-reviewed journal and presented at national and/or international conferences.

## Introduction

Menopause is a natural physiological transition marking the permanent cessation of menstruation, typically occurring between the ages of 45 and 55 years, and may also arise earlier through surgical, medical, or premature pathways ( National Institute for Health and Care Excellence, 2015). It is accompanied by a wide spectrum of symptoms that can meaningfully affect women’s functioning and quality of life. While vasomotor and psychological symptoms have historically received the most attention, musculoskeletal (MSK) symptoms are highly prevalent and increasingly recognised as a distinct feature of this period.

More than 70% of women experience MSK pain during the menopausal transition, and around 25% are disabled by it; this constellation of symptoms (arthralgia, myalgia, joint stiffness, reduced muscle mass and strength, and accelerated bone loss) has recently been described as the musculoskeletal syndrome of menopause ( Wright et al., 2024). Despite this prevalence, MSK pain in this group is often attributed to ageing alone, leaving it under-recognised and undertreated.

In parallel, mobile health (mHealth) applications have proliferated as a means of supporting women through menopause. mHealth refers to the use of mobile and wireless technologies, particularly smartphone applications, to support health and self-management, with symptom tracking and education among the core functions of menopause-related apps ( Sillence et al., 2025). The evidence underpinning these tools is, however, fragmented and of variable rigour, and concerns persist regarding their quality, usability, clinical validation, and effectiveness ( Paripoorani et al., 2023). Recent menopause-focused mHealth studies, including evaluations of Peppy ( Schei & Abernethy, 2024), Health & Her ( Andrews et al., 2023), Caria ( Duffecy et al., 2025), Mi Menopausia ( Coronado et al., 2025), and ZOE-related symptom-tracking tools ( Pounis et al., 2024), have examined symptom burden, quality of life, behavioural health outcomes, and symptom-monitoring approaches. Where MSK symptoms are represented within these studies, they are typically incorporated within broader symptom measures rather than assessed as standalone MSK outcomes. A prior review of menopause apps with an emphasis on osteoporosis ( Paripoorani et al., 2023) signalled limited attention to bone and MSK health, but no review has yet mapped how the broader published mHealth evidence base addresses the MSK symptoms of menopause.

The gap matters because symptom tracking is the principal mechanism by which mHealth tools could capture MSK symptoms, yet MSK is often represented only indirectly, embedded within composite menopause-symptom measures, or approached functionally through exercise and physical-activity components rather than through dedicated MSK assessment. Mapping whether and how the published evidence captures MSK symptoms is therefore necessary to identify where current research is weak. The scope of this review is restricted to published research evidence; a complementary review of the applications themselves is being conducted separately. Because the evidence base is heterogeneous and still emerging, a scoping review is the appropriate method for mapping the literature and identifying gaps for future research ( Arksey & O’Malley, 2005; Peters et al., 2024). Accordingly, this protocol describes a scoping review that will map menopause-related mHealth research, with particular attention to its coverage of MSK symptoms and whether they are assessed directly or only indirectly.

## Aims/objectives

The aim of this scoping review is to map and synthesise published research on mHealth applications designed for menopausal women, focusing on their purpose, functionality, and symptom-tracking capability, and on the extent and directness with which this research assesses or addresses MSK symptoms.

The specific objectives of the review are:

1. To map the extent, range, and nature of published research on mHealth interventions including a musculoskeletal focus, used by women during the menopausal transition.
2. To characterise the mHealth tools described in this research, including their key features, target users, intended outcomes, and symptom-tracking functionality.
3. To determine the extent to which this research assesses or addresses the musculoskeletal symptoms of menopause, distinguishing direct measurement (e.g., MSK-specific symptom tracking) from indirect coverage (e.g., MSK embedded within composite measures, or addressed through exercise/physical-activity components).
4. To identify where MSK symptom assessment remains underdeveloped in this literature, in order to inform future research.

## Methods

The overall conduct of this scoping review is informed by the Joanna Briggs Institute (JBI) methodological framework for scoping reviews ( Peters et al., 2024), building on the foundational approach of Arksey and O’Malley (2005). Reporting will follow the Preferred Reporting Items for Systematic Reviews and Meta-Analyses extension for Scoping Reviews (PRISMA-ScR; Tricco et al., 2018). This protocol is registered through its publication in HRB Open Research.

### Research question

The review will address the question: What does published research reveal about mHealth interventions for women during the menopausal transition, and to what extent does this research assess or address the MSK symptoms of menopause – whether directly through symptom tracking, or indirectly through composite symptom measures or exercise/physical-activity components?

### Study selection

For the purposes of this review, MSK symptoms encompass arthralgia, myalgia, joint stiffness, reduced muscle mass or strength, and bone-density-related changes (e.g., osteopenia or osteoporosis) arising during the menopausal transition. The registered title refers to musculoskeletal pain, but the review addresses this broader set of musculoskeletal symptoms, and the completed review will adopt the broader terminology. Eligibility will be structured using the JBI Population–Concept–Context (PCC) framework and is presented in full in Table 1. In brief, the review includes published research concerning adult women experiencing perimenopause, menopause, or postmenopause (Population) and patient-facing mHealth applications intended to support menopause, where the title or abstract indicates symptom monitoring or tracking with a direct or indirect MSK link (Concept), in any setting or country (Context). No comparator will be required for inclusion, and no study is excluded on the basis of reported outcomes alone. Eligible designs span quantitative, qualitative, and mixed-methods primary research, as well as app-development, participatory-design, usability, and feasibility studies. Only English-language studies of human populations are included, because the review had limited time and resources and no funding for translation. This is a limitation of the review. A separate grey-literature search will not be undertaken, so theses and non-peer-reviewed reports will not be systematically sought, because the review is scoped to published research. This is a further limitation. No restriction is placed on publication year. A detailed, study-level list of exclusions is provided in the extended data ( Buttenham et al., 2026).

**Table 1.** Inclusion and exclusion criteria structured by the JBI Population–Concept–Context (PCC) framework. The full intervention- and publication-level exclusion list is provided inthe extended data ( Buttenham et al., 2026).

Criterion	Inclusion	Exclusion
**Population**	Adult women experiencing perimenopause, menopause, or postmenopause, including natural, surgical, early, and premature menopause/primary ovarian insufficiency where menopause-related symptoms, health, or management are the focus.	Premenopausal populations without a menopause-related focus; pregnancy, postpartum, fertility, ovulation, or menstrual/period-tracking populations; male and non-human populations.
**Concept**	Published research describing, developing, testing, evaluating, or assessing patient-facing mobile health (mHealth) applications (including smartphone, self-management, and wearable-connected apps) intended to support menopausal women, where the title or abstract indicates symptom monitoring or tracking with a direct or indirect musculoskeletal (MSK) link. A composite menopause symptom measure that captures somatic/MSK items (e.g., the Menopause Rating Scale [ Heinemann et al., 2004] or Greene Climacteric Scale [ Greene, 1998]) satisfies this requirement as an indirect link. Where the only relevant functionality is exercise or physical activity, the study is eligible if oriented to strength or musculoskeletal benefit. Where it is unclear at title/abstract whether the symptoms tracked include an MSK dimension, the record is retained as a “maybe” and resolved at full text.	Non-digital or web-only interventions without a mobile application component; interventions where the digital tool is not patient-facing; applications offering only education or awareness with no symptom-monitoring or tracking dimension; symptom tracking confined to non-musculoskeletal symptoms (e.g., vasomotor or psychological symptoms only) with no MSK link; and activity tracking oriented to weight loss, calorie counting, or diet/metabolic goals rather than musculoskeletal/strength benefit (see the extended data for the full intervention-level exclusion list).
**Context**	Any setting or country. Published research evidence only. No comparator is required, and no study is excluded on the basis of reported outcomes alone. No restriction on publication year. English-language, human studies.	Studies addressing a related condition where menopause is not the primary focus (e.g., cancer, obesity, cardiovascular, pelvic-floor); non-English publications; non-human studies (see extended data).
**Study types**	Randomised controlled trials; non-randomised intervention studies; cohort, cross-sectional, qualitative, and mixed-methods studies; feasibility, usability, and pilot studies; participatory-design, requirements-gathering, and app-development/design studies. Conference papers where they report sufficient original research.	Editorials, commentaries, opinion pieces, news articles, company/funding announcements, press releases, and review protocols. Abstract-only publications and study protocols will be excluded.

Because the MSK relevance of a study often cannot be fully determined from its title and abstract alone, screening at this stage applies a symptom-signal gate. Title and abstract screening will be carried out independently by two reviewers, and a record will be carried through to full-text review only where it indicates all three of the following: an mHealth application; a menopause focus; and either symptom monitoring or recording that could relate to MSK symptoms, or fitness functionality involving MSK-oriented strength or exercise tracking. Because it is frequently difficult to judge from a title and abstract alone whether symptom tracking captures MSK symptoms, records in which an MSK dimension is suspected or cannot be ruled out will be carried through to full text, whereas records in which the symptom tracking is clearly non-musculoskeletal (for example, hot flushes or other vasomotor symptoms only) will not be. Conflicts at the title and abstract stage will be discussed and resolved by agreement between the two reviewers.

### Identifying relevant studies

The search strategy was developed and refined by the research team and combines controlled vocabulary (where available) with free-text title/abstract terms across two concept blocks: menopause and mHealth/digital health. Six bibliographic databases will be searched: Embase, Web of Science, Scopus, PubMed/MEDLINE, CINAHL, and IEEE Xplore. Reference lists of included studies and relevant reviews will be hand-searched to identify additional eligible records. Searches are restricted to English-language, human studies, with no restriction on publication year. A representative search strategy (PubMed/MEDLINE) is provided in Table 2, and the full database-specific strategies are provided in the extended data ( Buttenham et al., 2026). Supplementary musculoskeletal-focused searches were also conducted to scope the volume of MSK-specific literature; these are reported in the extended data.

**Table 2.** Representative search strategy (PubMed/MEDLINE). Equivalent strategies were adapted for Embase, Web of Science, Scopus, CINAHL, and IEEE Xplore.

Concept block	Search terms (PubMed/MEDLINE, illustrative)
**Population**	“Menopause”[Mesh] OR menopause [tiab] OR menopausal [tiab] OR perimenopause [tiab] OR perimenopausal [tiab] OR postmenopause [tiab] OR postmenopausal [tiab] OR climacteric [tiab] OR “menopausal transition”[tiab]
**AND Concept**	“Mobile Applications”[Mesh] OR “Telemedicine”[Mesh] OR “Cell Phone”[Mesh] OR mHealth [tiab] OR “mobile health”[tiab] OR eHealth [tiab] OR “digital health”[tiab] OR “mobile app*”[tiab] OR “smartphone app*”[tiab] OR “health app*”[tiab] OR smartphone [tiab] OR “mobile technolog*”[tiab] OR telehealth [tiab] OR telemedicine [tiab] OR “app-based”[tiab] OR “digital intervention*”[tiab]

### Charting the data

Records will be managed in Covidence. Title and abstract screening and subsequent full-text review will each be conducted independently and in duplicate by two reviewers (OB and KMcC). At full text, a study will be included only where it satisfies the criteria set out in the master extraction form, namely an mHealth application, a menopause focus, and MSK-related information; studies that do not meet these criteria will be excluded at full text. Conflicts are resolved by discussion and agreement between the two reviewers, or third author consultation (KC), and reasons for exclusion at full text will be recorded and reported in the PRISMA-ScR flow diagram in the completed review.

Data are charted using a standardised extraction form, piloted on a sample of included studies and refined as needed. For each included study, the form captures the following:

• **Study identification and bibliographic details** — study and app identifiers, author, year, full citation, journal, DOI, and source database.
• **Study context** — country, setting, aim, and any stated research questions.
• **Design** — study design, methodological detail, evidence level, comparator, and control condition.
• **Participants** — sample size, population, age, menopause status, eligibility criteria, recruitment, follow-up, and attrition.
• Intervention — application description, symptom-tracking functionality (including whether tracking covers MSK-relevant symptoms), duration, intensity, engagement metrics, and adherence.
• **Outcomes** — primary and secondary outcomes, outcome category, and measurement tools.
• **MSK focus (the core review domain)** — the MSK-directness classification (direct-primary, direct-explicit-secondary, indirect-composite, or indirect-exercise/physical-activity), which MSK symptoms are captured (e.g., joint pain, muscle, bone/osteoporosis), the measurement tool and approach where applicable, dimensions assessed (e.g., pain intensity or pain interference), assessment frequency where reported, whether MSK is explicitly measured, whether it is analysed separately, and a summary of MSK findings. This classification will be applied at full text (see below).
• **Other symptoms** — non-MSK symptoms assessed and the tools used.
• **Data collection** — assessment frequency, data-collection method, and real-world-use classification.
• **Statistical analysis** — methods, effect sizes, significance, and confidence intervals.
• **Results** — key findings, clinical implications, and proposed mechanisms.
• **Quality and limitations** — author-reported limitations and reviewer-noted methodological considerations, including aspects such as sample size, attrition, and completeness of reporting, recorded descriptively to support interpretation of the evidence base.
• **Funding and ethics** — funding source, declared conflicts of interest, and ethics approval.
• **Generalisability** — applicability to broader populations and equity or diversity considerations.
• **Acceptability and implementation** — technology acceptability, usability, and engagement insights (organised through the Technology Acceptance Model; Davis, 1989), charted as a secondary, descriptive dimension used to characterise the applications rather than as a primary review aim and reported descriptively, together with implementation barriers and facilitators.
• **Synthesis and review coding** — consistency with other studies, contradictions, relevance to the review, directness to the MSK objective, and data-extraction confidence.

Consistent with scoping review methodology, methodological quality will not be used to determine inclusion, and no study will be included or excluded on the basis of its risk of bias. No formal methodological quality or risk-of-bias appraisal will be undertaken, in line with scoping review methodology. Author-reported limitations will be charted, and the review team will additionally record brief descriptive notes on relevant methodological considerations, such as sample size, attrition, and completeness of reporting, to provide context for the narrative synthesis. These notes will not constitute a formal risk-of-bias assessment. The review does not appraise applications using app-rating instruments such as the Mobile App Rating Scale, as this protocol concerns the published research evidence rather than the applications themselves.

To address the central objectives, each included study will be coded at full text for how directly it assesses musculoskeletal symptoms, using the four categories in Table 3. These run from direct measurement, where MSK is assessed as a primary outcome or as a distinct secondary outcome, to indirect coverage, where MSK is captured only within a broader composite symptom measure or addressed through exercise or physical-activity content.

**Table 3.** Classification of musculoskeletal (MSK) coverage in included studies, applied at full text.

Classification	Definition	Example
**Direct – primary**	MSK symptom(s) are a primary outcome, measured with an MSK-specific instrument.	Validated joint-pain or arthralgia scale used as the primary endpoint.
**Direct – explicit secondary**	MSK directly measured but not the primary outcome; reported as a distinct item, subscale, or outcome.	A dedicated joint- or muscle-symptom item or subscale reported separately.
**Indirect – composite**	MSK embedded within a broader composite menopause-symptom measure and not analysed separately.	“Joint aches” within a multi-symptom physical domain; MRS somatic subscale.
**Indirect – exercise/physical activity**	No MSK symptom measure, but MSK addressed functionally through exercise, mobility, or physical-activity components.	App mobility/exercise module or physical-activity outcomes without MSK symptom tracking.

### Collating and summarising results

A narrative synthesis will be undertaken, supported by descriptive statistics and frequency tables, and organised around the review objectives: (i) the extent, range, and characteristics of menopause-related mHealth research; (ii) the design and intended use of the tools studied; (iii) how directly MSK is covered (direct-primary, direct-secondary, indirect-composite, or indirect-exercise); and (iv) the gaps and weaknesses identified in how MSK symptoms are captured. Where appropriate, thematic summaries and illustrative examples will characterise patterns across studies. Results will be reported in accordance with PRISMA-ScR, including a flow diagram of the selection process and summary tables of charted data.

## Discussion

This protocol describes the rationale and planned conduct of a scoping review examining how published mHealth research addresses the MSK symptoms of menopause. Making the protocol publicly available provides a transparent record of the planned methods and allows any deviations from the original approach to be clearly identified. The MSK syndrome of menopause is highly prevalent yet under-recognised, and digital tools are increasingly positioned as scalable supports; mapping whether (and how directly) existing research captures MSK symptoms will clarify an important and currently uncharacterised gap. The review is designed to characterise the current evidence and identify areas that remain underdeveloped, including whether MSK symptoms are directly assessed or primarily represented indirectly. Beyond identifying research gaps, the findings may inform the future design of menopause-related mHealth applications, for example by encouraging more explicit MSK symptom tracking, and support clinical practice by drawing attention to a prevalent but under-recognised symptom cluster.

Anticipated limitations include restriction to English-language records; the heterogeneity and variable rigour of the underlying evidence, which constrains comparison across studies, as well as the fact that scoping reviews do not formally appraise study quality. These limitations are characteristic of scoping reviews in fast-moving mHealth fields and will be acknowledged transparently in the completed review.

## Conclusion

This review will document how MSK symptoms are incorporated into menopause-related mHealth research, and whether they are assessed directly or only indirectly within broader symptom frameworks. Its findings should help direct future primary research and the design of digital tools that give greater attention to the MSK symptoms of menopause.

### Study status

The review has commenced. Searches have been conducted and screening is underway. It is being undertaken as an eight-week project (planned start June 2026) supported by a HRB Summer Studentship under academic supervision at the University of Limerick.

###  Dissemination

An article reporting the results of the scoping review will be submitted to a peer-reviewed scientific journal and presented at relevant national and/or international conferences. A project report and a draft manuscript will be prepared as part of the funded scholarship outputs.

## Ethics and consent

Ethical approval and consent were not required. This scoping review uses only published, publicly available sources and does not involve human participants, their data, or animals.

## Declaration of generative AI use

During the preparation of this work, the authors used Claude Opus 4.5 for language editing and suggestions, formatting, and structuring the manuscript to the journal’s requirements. The authors reviewed and edited all content and take full responsibility for the content of the publication.

## Data Availability

No underlying data are associated with this article, as it reports a protocol and generates no primary data. The materials supporting the protocol — the eligibility criteria, database search strategies, and data-charting framework — are provided within the article and as extended data (see Extended data and Reporting guidelines below). The full study-level exclusion list and the complete database search strategies are provided as extended data, deposited in Zenodo: https://doi.org/10.5281/zenodo.21285615 ( Buttenham et al., 2026). Data are available under the terms of the Creative Commons Attribution 4.0 International license (CC-BY 4.0). Reporting of this protocol and of the completed review follows the PRISMA Extension for Scoping Reviews (PRISMA-ScR). A completed PRISMA-ScR checklist will be provided with the final review article.
