# Supplementary material for: mHealth Applications in the Assessment of Musculoskeletal Pain in Menopausal Women: Protocol for a Scoping Review
Source: HRB Open Res. 2026 Sep 22;9:80. Originally published 2026 Jul 29. [Version 2] doi: 10.12688/hrbopenres.14515.2 (PMC13627943; doi:10.12688/hrbopenres.14515.2)
Supplement: Peer review history

# Reviewer report: Reviewer response for version 2

**DOI:** 10.21956/hrbopenres.16050.r58040

**Published:** 2026-09-30

**Authors:** Nigel R. Armfield

**Affiliations:** 1 The University of Queensland, Brisbane, Australia

Amendments reviewed; no further comments.

Is the study design appropriate for the research question?

Yes

Is the rationale for, and objectives of, the study clearly described?

Yes

Are sufficient details of the methods provided to allow replication by others?

Yes

Are the datasets clearly presented in a useable and accessible format?

Not applicable

Reviewer Expertise:

Epidemiology, Pain, Digital Health

I confirm that I have read this submission and believe that I have an appropriate level of expertise to confirm that it is of an acceptable scientific standard.

# Reviewer report: Reviewer response for version 1

**DOI:** 10.21956/hrbopenres.16000.r56969

**Published:** 2026-09-01

**Authors:** Nigel R. Armfield

**Affiliations:** 1 The University of Queensland, Brisbane, Australia

Thank you for the opportunity to review this interesting paper. It is well written and was a pleasure to read. The work proposed by the protocol is valuable and addresses an important topic of women’s health.

 The rationale and research gap is well justified: mHealth may benefit women during menopausal transition, however, the extent to which applications *explicitly/directly* capture musculoskeletal *(MSK) symptoms of menopause*, rather than  composite measures that *incorporate* MSK symptoms indirectly is unclear.

 The evidence is unclear because no previous reviews have been conducted; by addressing this gap, this review described in the protocol will provide an evidence map that will influence both future research and mHealth design. This type of evidence is essential to ensure that future mHealth applications are optimally designed to address the needs of the women they are intended to help. More generally, the proposed review is an example of the type of work that is needed to overcome the (unfortunately common) waste associated with poor digital health design and subsequent poor adoption

 The justification for a scoping review to map the literature is well made and appropriate, as are the procedures and reporting. The authors have developed their protocol by reference to the JBI framework for conducting scoping reviews and intend to report using the PRISMA Extension for Scoping Reviews (PRISMA-ScR). The search strategy and sources appear comprehensive.

 I have some comments / suggestions. While they are of a minor nature, I recommend addressing them.

 1. The title specifically mentions "MSK *pain*" whereas elsewhere the paper largely talks about "MSK *symptoms*" (of which one symptom is obviously pain) but pain does not appear prominently in the list of dot points for the charting. I was curious whether the review would map which pain measures have been reported (e.g. *pain intensity*, *pain interference* etc), how they have been measured, and the frequency of measurement etc.

 2. Use of the Technology Acceptance Model (TAM) to report acceptability / usability / engagement - I wondered why this was included when assessment of these aspects is not an explicit aim of the review. If it is important, then I suggest adding it as an aim / research question. If it is not central then it could perhaps be removed without causing harm.

 3. The review includes studies published in the English language. While common, some justification could be provided for this.

 4. Scoping reviews typically include the grey literature. In this case, it may not be beneficial/practical to include it, but again a statement could be provided to justify the exclusion.

 5. The methods section changes tense between future and present; since it's a protocol I suggest adjusting to future tense.

 6. Risk-of-bias (RoB) assessment - this is uncommon in a mapping study, and I was a little unclear about whether the authors will (a) only provide a descriptive RoB assessment where included studies have provided their own RoB, or (b) will provide a descriptive RoB assessment for all included studies. Perhaps it could be clarified, and a justification for conducting RoB assessment added.

 7. Suggest citing the JBI Manual for Evidence Synthesis, Chapter 10 which was last updated in 2024 (rather than the Peters 2020 reference you currently have). The relevant citation is at the top of this page: https://doi.org/10.46658/JBIMES-24-09).

Is the study design appropriate for the research question?

Yes

Is the rationale for, and objectives of, the study clearly described?

Yes

Are sufficient details of the methods provided to allow replication by others?

Yes

Are the datasets clearly presented in a useable and accessible format?

Not applicable

Reviewer Expertise:

Epidemiology, Pain, Digital Health

I confirm that I have read this submission and believe that I have an appropriate level of expertise to confirm that it is of an acceptable scientific standard, however I have significant reservations, as outlined above.

# Response

**Published:** 2026-09-06

**Authors:** Olivia Buttenham

**Affiliations:** Medicine, University of Limerick School of Medicine, Limerick, County Limerick, Ireland

Response to reviewer comments

**Manuscript:** mHealth Applications in the Assessment of Musculoskeletal Pain in Menopausal Women: Protocol for a Scoping Review

 Thank you for reviewing our protocol and for the helpful comments. We have worked through each one below. Where a change also shows up in the completed review, we say so.

**1. The title mentions “MSK pain” but the paper mostly refers to “MSK symptoms”. Would the review map which pain measures are reported, and how they are measured?**

 You are right that the terms did not match. The protocol title is already registered, so we cannot change it now, but we have added a note in the protocol to make clear that the review covers the broader set of musculoskeletal symptoms and not pain alone, and that the completed review will use the broader term “musculoskeletal health outcomes”. The body of the protocol already defines the scope this way, covering joint pain, muscle pain, stiffness, weakness, reduced physical function and bone health. On measurement, the review will record how each outcome is captured, measured and reported, including the specific instrument and how often it is used where a study gives that detail, so the point you raised is covered. The charting form now names these explicitly, including pain intensity, pain interference, and how often assessment was done where reported.

**2. Why use the Technology Acceptance Model (TAM) when acceptability, usability and engagement are not stated aims?**

 A fair question. TAM was pre-registered as the framework for the acceptability and implementation charting category, so we kept it, but we added wording to pin down its role. These items will be charted as a secondary descriptive dimension to help describe the apps, not as a primary aim, and will be reported descriptively rather than as an evaluation. Dropping a pre-registered element would have meant another deviation, so clarifying felt like the cleaner choice.

**3. The English-language restriction could be justified.**

 Agreed. We added a short justification. English-only records were used because the project had limited time and resources and no funding for translation. This now sits in the limitations.

**4. Grey literature is usually included in scoping reviews, and the exclusion could be justified.**

 We added a line on this too. A separate grey-literature search will not be undertaken, so theses and non-peer-reviewed reports will not be systematically sought, since the review is scoped to published research identified through the databases and reference-list searching. Indexed conference papers that report sufficient original research can still qualify, so this does not conflict with the eligibility criteria. It is listed as a limitation.

**5. The methods switch between present and future tense, and future tense is suggested for a protocol.**

 Thank you for catching that. We have put the prospective review procedures in the Methods into the future tense, for example screening, charting, coding, and conflict resolution. A few statements stay in the past tense on purpose, because they describe steps that have already happened, such as developing the search strategy and running the searches. The Study status section notes that the searches are done and screening is underway, so forcing those verbs into the future would misstate the timeline.

**6. Risk of bias: will the descriptive assessment cover only studies that report their own, or all studies? Please clarify and justify.**

 We have reconciled this in the protocol. No formal methodological quality or risk-of-bias appraisal will be undertaken, in keeping with scoping review methodology, and no study will be included or excluded on that basis. To avoid implying a formal appraisal, we have removed the term risk-of-bias judgement. Instead, author-reported limitations will be charted, and the review team will record brief descriptive notes on methodological points such as sample size, attrition and completeness of reporting, to give context to the synthesis. These notes will not be a formal risk-of-bias assessment. The protocol now matches this.

**7. Suggest citing the JBI Manual for Evidence Synthesis, Chapter 10 (2024) rather than Peters 2020.**

 Changed. The citation is now the JBI Manual for Evidence Synthesis, Chapter 10 (Peters et al., 2024), https://doi.org/10.46658/JBIMES-24-09.

 Thanks again for the comments. They have made the protocol clearer.

# Reviewer report: Reviewer response for version 1

**DOI:** 10.21956/hrbopenres.16000.r57307

**Published:** 2026-08-18

**Authors:** Camille Cronin

**Affiliations:** 1 University of Essex, Colchester, UK

Thank you to the authors for the opportunity to review this scoping review protocol. This is a timely and relevant area of research, addressing the growing use of mHealth applications in menopause and the relatively under-recognised issue of musculoskeletal symptoms.

 Summary of Paper

 The paper presents a protocol for a scoping review examining how mHealth applications used during menopause assess and address musculoskeletal (MSK) symptoms. Using JBI methodology and PRISMA-ScR guidance, the review will map the available evidence, with particular attention to whether MSK symptoms are measured directly or captured indirectly within broader symptom measures or physical activity components.

 Overall, the rationale, aim and objectives are clearly presented and the scoping review design is appropriate to the research question.

 The protocol is well structured and follows JBI methodology and PRISMA-ScR guidance. The eligibility criteria, search strategy, screening process, data charting and proposed synthesis are described in sufficient detail, with additional search materials provided as extended data. The proposed classification of direct and indirect MSK coverage is particularly helpful in providing a clear focus for the review.

 I have only a few minor points for the authors to consider:

- The protocol states that a descriptive risk-of-bias judgement will be recorded, while also acknowledging that formal quality appraisal is not normally undertaken within a scoping review. It would be helpful to clarify how this judgement will be made, including whether a particular framework or criteria will be used, and how it will contribute to the synthesis. Alternatively, the authors may wish to reconsider whether this element is required.
- The Technology Acceptance Model is proposed to organise findings relating to acceptability, usability and engagement. A brief rationale for the inclusion of this framework and how it contributes to the central MSK-focused objectives would strengthen the protocol.
- The authors include anticipated findings suggesting that MSK symptoms are likely to be less frequently and more indirectly represented. As the purpose of a scoping review is to map the available evidence, the authors may wish to consider whether stating anticipated findings is necessary within the protocol.

Overall, this is a clearly presented and methodologically appropriate protocol requiring some minor amendments.

Is the study design appropriate for the research question?

Yes

Is the rationale for, and objectives of, the study clearly described?

Yes

Are sufficient details of the methods provided to allow replication by others?

Yes

Are the datasets clearly presented in a useable and accessible format?

Not applicable

Reviewer Expertise:

Women's health - menopause

I confirm that I have read this submission and believe that I have an appropriate level of expertise to confirm that it is of an acceptable scientific standard.

# Response

**Published:** 2026-09-15

**Authors:** Olivia Buttenham

**Affiliations:** Medicine, University of Limerick School of Medicine, Limerick, County Limerick, Ireland

Thank you for reviewing our protocol and for your positive assessment. We have addressed your three points below.

 Risk of bias. We agree this needed to be clearer, and this point was also raised by the other reviewer, so we have addressed both together. No formal methodological quality or risk-of-bias appraisal will be undertaken, which is consistent with scoping review methodology, and no study will be included or excluded on that basis. We have removed the term risk-of-bias judgement to avoid implying a formal appraisal. Instead, the review team will record brief descriptive methodological notes, such as sample size, attrition, and completeness of reporting, to provide context for the synthesis. These will not constitute a formal risk-of-bias assessment.

 Technology Acceptance Model. We have added a brief rationale. TAM was pre-registered as the framework for the acceptability and implementation charting category. We have clarified that acceptability, usability, and engagement are charted as a secondary descriptive dimension used to characterise the applications rather than as a primary aim, and that they are reported descriptively. This keeps the focus on the central musculoskeletal objectives while retaining useful descriptive context about the apps.

 Anticipated findings. We take this point. Because the purpose of a scoping review is to map the available evidence rather than to test an expectation, we have removed the anticipated-findings statement so that the aims and objectives stand on their own.

 Thank you again for your time and for these constructive comments.
